# *N*-eicosapentaenoyl-ethanolamine decreases the proliferation of psoriatic keratinocytes in a reconstructed psoriatic skin model

**DOI:** 10.1038/s41598-023-39185-4

**Published:** 2023-07-26

**Authors:** Mélissa Simard, Andréa Tremblay, Sophie Morin, Geneviève Rioux, Nicolas Flamand, Roxane Pouliot

**Affiliations:** 1 Centre de Recherche en Organogénèse Expérimentale de l’Université Laval/LOEX, Axe médecine régénératrice, Centre de recherche du CHU de Québec-Université Laval, Québec, QC G1J 1A4 Canada; 2 Faculté de Pharmacie de l’Université Laval, Québec, QC Canada; 3 Centre de recherche de l’Institut universitaire de cardiologie et de pneumologie de Québec, Département de médecine, Faculté de médecine, Université Laval, Québec, QC G1V 4G5 Canada; 4 Canada Excellence Research Chair On the Microbiome-Endocannabinoidome Axis in Metabolic Health (CERC-MEND), Université Laval, Québec, QC G1V 0A6 Canada

**Keywords:** Skin diseases, Inflammation, Biomedical engineering

## Abstract

Psoriasis is an inflammatory skin disease that is characterized by keratinocyte hyperproliferation, abnormal epidermal differentiation and dysregulated lipid metabolism. Some lipid mediators of the *N*-acylethanolamines (NAEs) and monoacylglycerols (MAGs) can bind to cannabinoid (CB) receptors and are referred to as part of the endocannabinoidome. Their implication in psoriasis remains unknown. The aim of the present study was to characterize the endocannabinoid system and evaluate the effects of n-3-derived NAEs, namely *N*-eicosapentaenoyl-ethanolamine (EPEA), in psoriatic keratinocytes using a psoriatic skin model produced by tissue engineering, following the self-assembly method. Psoriatic skin substitutes had lower *FAAH2* expression and higher *MAGL*, *ABHD6* and *ABHD12* expression compared with healthy skin substitutes. Treatments with alpha-linolenic acid (ALA) increased the levels of EPEA and 1/2-docosapentaenoyl-glycerol, showing that levels of n-3 polyunsaturated fatty acids modulate related NAE and MAG levels. Treatments of the psoriatic substitutes with 10 μM of EPEA for 7 days resulted in decreased epidermal thickness and number of Ki67 positive keratinocytes, both indicating decreased proliferation of psoriatic keratinocytes. EPEA effects on keratinocyte proliferation were inhibited by the CB_1_ receptor antagonist rimonabant. Exogenous EPEA also diminished some inflammatory features of psoriasis. In summary, n-3-derived NAEs can reduce the psoriatic phenotype of a reconstructed psoriatic skin model.

## Introduction

Psoriasis is an autoimmune inflammatory disease that most often manifests as well-demarcated erythematous papules and plaques covered with silvery scales. Psoriasis is associated with an accentuated proliferation of epidermal keratinocytes leading to a massive thickening of the epidermis^1,2^. Another important histological hallmark of the disease is an atypical cell differentiation in the skin accompanied by parakeratosis, with retention of the nuclei in the keratinocytes of the stratum corneum^3^. Some specific proteins are also expressed concomitantly with psoriatic keratinocyte proliferation, such as keratin 17 and psoriasin (S100A7). The exact cause of psoriasis is poorly understood but it certainly involves the immune stimulation of keratinocytes in which T cells seem to play a central role^4^.

Keratinocyte proliferation and differentiation are processes that are highly regulated by various signaling systems, involving a large number of bioactive lipids derived from polyunsaturated fatty acids (PUFAs), which include prostanoids, hydroxy fatty acids, leukotrienes and endocannabinoids^5,6^. On the one hand, some n-6 PUFA lipid mediators, such as leukotriene B_4_ (LTB_4_) and prostaglandin E_2_ (PGE_2_), participate in the secretion of cytokines and the recruitment of inflammatory cells in psoriatic skin^7^. On the other hand, lipid mediators derived from n-3 PUFAs and endocannabinoids were shown to exert pro-resolving and/or anti-inflammatory effects^8–10^. The endocannabinoids *N*-arachidonoyl-ethanolamine (AEA) and 2-arachidonoyl-glycerol (2-AG) contain an arachidonic acid (n-6 PUFA) moiety and are endogenous ligands of the cannabinoid type-1 (CB_1_) and -2 (CB_2_) receptors^11–13^. AEA is part of the large family of *N*-acyl-ethanolamines (NAEs) while 2-AG is a monoacylglycerol (MAG)^14^. Over the past 15 years, many NAEs and MAGs containing other 16-plus carbon fatty acids have been shown to share some effects similar to endocannabinoids, and thus have been grouped under the term endocannabinoidome^15^. The congeners of AEA and 2-AG that are part of the endocannabinoidome notably include NAEs and MAGs containing n-3 PUFAs such as eicosapentaenoic acid (EPA) and docosahexaenoic acid (DHA). The EPA- and DHA-containing NAEs are *N*-eicosapentaenoyl-ethanolamine (EPEA) and *N*-docosahexaenoyl-ethanolamine (DHEA) respectively^16,17^. In addition to being involved in skin and other tissue homeostasis, the endocannabinoid system holds a strong therapeutic potential for many inflammatory diseases, as the activation of CB_1_ and CB_2_ receptors modulates inflammatory responses, at least in mice^18–20^.

Although the involvement of the endocannabinoid system in skin homeostasis appears to be increasingly important, the therapeutic potential of endocannabinoids for the treatment of skin diseases such as psoriasis remains unclear. In this regard, tissue-engineered human psoriatic skin models have been shown to be efficient pre-clinical tools for assessing the biological activities of potential drugs^21,22^. Indeed, these models display hyperproliferative psoriatic keratinocytes, disturbed epidermal differentiation, elevated pro-inflammatory cytokine and lipid mediator levels and dysregulated gene expression characteristic of psoriatic skin^23–25^. In addition, tissue-engineered psoriatic models were shown to respond to current available treatments for psoriasis, including tazarotene, retinoic acid and anti-interleukin-17^26–28^. In a previous study, we showed that psoriatic skin substitutes produced using culture media supplemented with the n-3 PUFA alpha-linolenic acid (ALA) led to a decreased keratinocyte proliferation and a more complete terminal differentiation compared with untreated controls, supporting some anti-inflammatory properties of ALA in psoriasis^29^. In the present study we expanded these analyses to investigate whether the effects associated with n-3 PUFA supplementation could be attributed to increased metabolism into n-3-derived NAEs and MAGs. More specifically, we compared the levels of endocannabinoids and related mediators of healthy and psoriatic skin substitutes using liquid chromatography tandem mass spectrometry (LC–MS/MS) and established whether the n-3 PUFA therapeutic effects on reconstructed psoriatic skin could be exerted, in part, through the endocannabinoidome.

## Results

### The endocannabinoid system is altered in psoriatic skin substitutes

**Figure 1:**
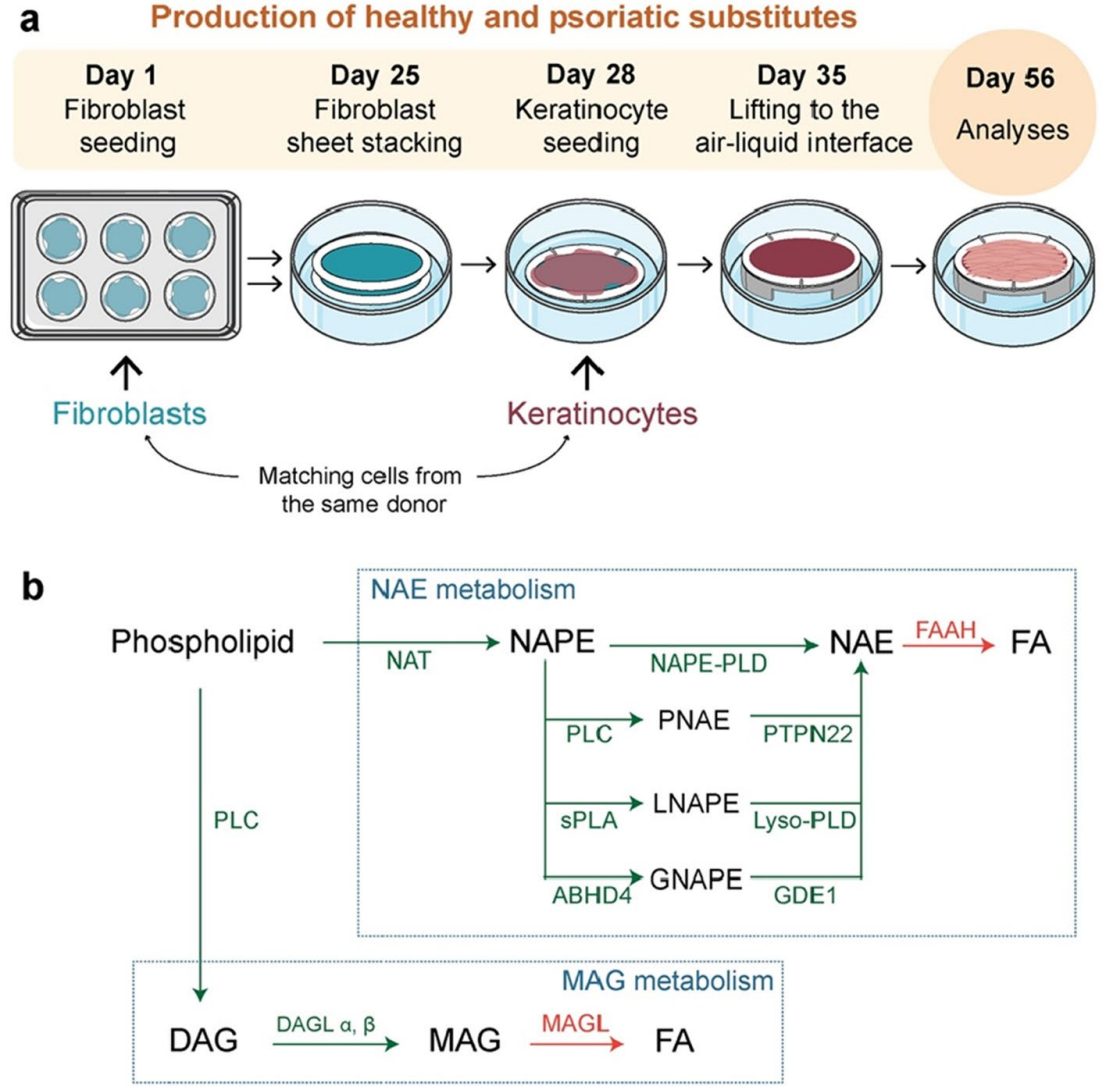
Identification of proteins of the endocannabinoid system in healthy and psoriatic substitutes. (**a**) Schematic overview of skin substitute reconstruction according to the self-assembly method. (**b**) Schematic overview of the synthesis and degradation pathways of MAGs and NAEs and related receptor signaling. *ABHD4* Abhydrolase domain-containing protein 4, *CB1-2* cannabinoid receptor 1–2, *DAG* diacylglycerol, *DAGL* diacylglycerol lipase, *FAAH* fatty-acid amide hydrolase 2, *GDE1* glycerophosphodiesterase 1, *HS* healthy substitute, *MAG* monoacylglycerol, *MAGL* monoacylglycerol lipase, *NAE* *N*-acyl-ethanolamine, *NAPE* *N*-acyl-phosphatidylethanolamine, *NAT* arylamine *N*-acetyltransferase, *PLC* phospholipase C, *PLD* phospholipase D, *PS* psoriatic substitute, *PTPN22* Tyrosine-protein phosphatase non-receptor type 22, *sPLA* secretory phospholipase A.

Tissue-engineered skin substitutes were produced according to the self-assembly method using cells isolated from biopsies of either healthy donors (HS^-^) or donors with psoriasis (PS^-^) (Fig. 1a). The expression of gene-encoding proteins linked to the endocannabinoidome was investigated in HS^-^ and PS^-^ after 56 days of culture using gene profiling to identify whether it was altered in psoriatic epithelial cells (Table 1). NAE biosynthesis can occur through 4 different pathways (Fig. 1b). The gene-encoding proteins of the classical NAE biosynthetic pathway, namely *NAT* and *NAPEPLD,* were both expressed in the skin substitutes (Table 1). Moreover, the expression of *ABHD4*, *GDE1*, *PLA2*, *ENPP2* and *GDPD3* was detected in the skin substitutes, indicating that both the sPLA/Lyso-PLD and the ABH4/GDE1 pathways might be relevant in the skin substitutes as well (Table 1). Of note, the exact PLA2 among the PLA2 family responsible for NAE biosynthesis have not been determined and therefore other PLA2 could be involved. On the other hand, the expression of *PLCB1*, *PLCB2*, *PLCB3*, *PLCB4* and *PTPN22* was not detected in the skin substitutes under our culture conditions, suggesting that this pathway is unlikely to be involved in the biosynthesis of NAEs in the skin substitutes (Table 1). Interestingly, a sixfold increase in the expression of *ENPP2* was measured in the PS^-^ samples, compared with HS^-^, thus showing that Lyso-PLD could be involved in altered NAE signaling in psoriasis (Table 1). Other genes were not significantly altered in PS^-^ as compared with HS^-^ (Table 1). Transcriptomic analyses revealed low expression of genes (*FAAH1* and *FAAH2*) encoding proteins involved in the degradation of NAEs and high expression of genes (*MAGL*, *ABHD6* and *ABHD12*) encoding proteins involved in the degradation of MAGs (Table 1). In addition, *FAAH2* expression was lower in PS^−^ than in HS^-^, while the expression of all genes (*MAGL*, *ABHD6* and *ABHD12*) encoding MAG hydrolases was higher in PS^-^ than in HS^-^ (Table 1). Among the genes encoding receptors related to the endocannabinoidome, the expression of *CNR1*, *TRPV1* and *GPR119* was detected in the skin substitutes, showing that NAE and MAG signaling in the skin substitutes may be mediated through these receptors.

**Table 1:** Expression of gene-encoding proteins involved in the synthesis and the degradation of endocannabinoids.

eCB-like	Gene symbol	Gene name	Linear signal HS^−^	Linear signal PS^−^	Fold change (PS^−^/HS^−^)
NAE synthesis	*NAT1*	Arylamine *N*-acetyltransferase 1	297	316	1.1
	*NAT2*	Arylamine *N*-acetyltransferase 2	ND	ND	NA
	*NAPEPLD*	*N*-acyl-phosphatidylethanolamine-hydrolyzing phospholipase D	203	338	1.7
	*ABHD4*	Abhydrolase domain-containing protein 4	670.3	1066	1.1
	*GDE1*	Glycerophosphodiester phosphodiesterase 1	1591	3098	1.9
	*PLCB1*	1-Phosphatidylinositol 4,5-bisphosphate phosphodiesterase b-1	ND	ND	NA
	*PLCB2*	PLCB2 protein; 1-phosphatidylinositol 4,5-bisphosphate phosphodiesterase beta-2	ND	ND	NA
	*PLCB3*	1-Phosphatidylinositol 4,5-bisphosphate phosphodiesterase beta-3	ND	ND	NA
	*PLCB4*	Phospholipase C, beta 4; 1-phosphatidylinositol 4,5-bisphosphate phosphodiesterase beta-4	ND	ND	NA
	*PTPN22*	Tyrosine-protein phosphatase non-receptor type 22	ND	ND	NA
	*ENPP2*	Ectonucleotide pyrophosphatase/phosphodiesterase family member 2	116	707	6.1
	*GDPD1*	Glycerophosphodiester phosphodiesterase domain-containing protein 1	ND	ND	NA
	*GDPD3*	Glycerophosphodiester phosphodiesterase domain-containing protein 3	623	376	0.6
MAG synthesis	*DAGLA*	Diacylglycerol lipase alpha	ND	ND	NA
	*DAGLB*	Diacylglycerol lipase beta	ND	ND	NA
NAE degradation	*FAAH*	Fatty-acid amide hydrolase 1	ND	ND	NA
	*FAAH2*	Fatty-acid amide hydrolase 2	250.5	58.6	0.2*
MAG degradation	*MGLL*	Monoacylglycerol lipase	1726.7	3780	2.2*
	*ABHD6*	Monoacylglycerol lipase ABHD6	553	1349	2.4*
	*ABHD12*	Monoacylglycerol lipase ABHD12	230	596	2.6*
Receptors	*CNR1*	Cannabinoid receptor 1	186	460	2.5*
	*CNR2*	Cannabinoid receptor 2	ND	ND	NA
	*TRPV1*	Transient receptor potential cation channel subfamily V member 1	108.6	109.9	1.0
	*TRPV4*	Transient receptor potential cation channel subfamily V member 4	ND	ND	NA
	*GPR18*	G protein-coupled receptor 18	ND	ND	NA
	*GPR119*	Glucose-dependent insulinotropic receptor	99.2	118.7	1.2
	*GPR55*	G-protein-coupled receptor 55	ND	ND	NA

*eCB* endocannabinoid, *HS* healthy substitute, *MAG* monoacylglycerol, *NA* not applicable, *NAE* *N*-acyl-ethanolamine, *ND* not detected, *PS* psoriatic substitute.

**Figure 2:**
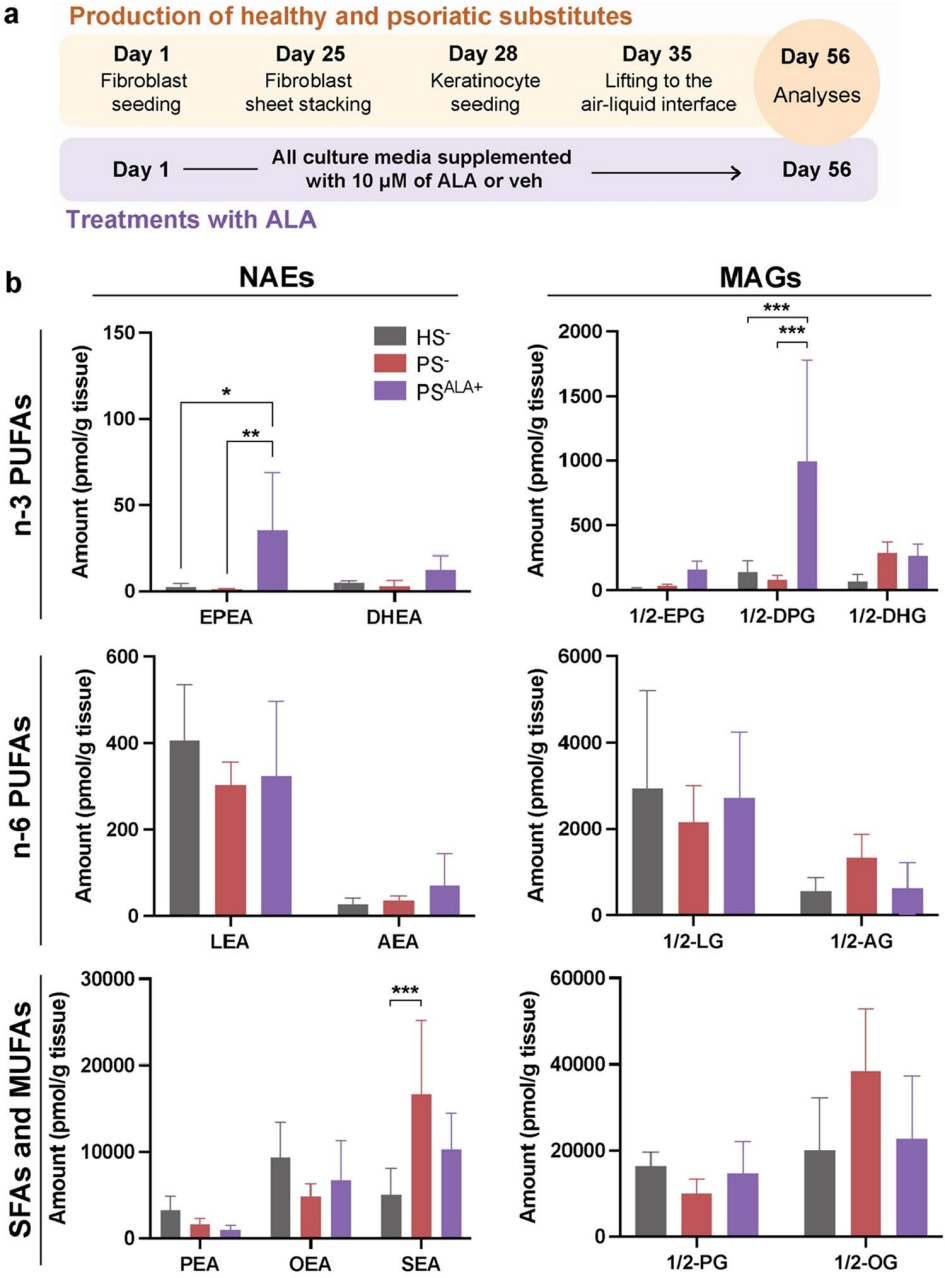
Biosynthesis of NAEs and MAGs in healthy and psoriatic skin substitutes after ALA treatment. (**a**) Schematic overview of skin substitute reconstruction according to the self-assembly method treated with either 10 μM ALA or the vehicle. (**b**) Amount of NAEs and MAGs in HS^−^, PS^−^ and PS produced with culture media supplemented with 10 μM ALA (PS^ALA+^), as determined by LC–MS/MS. Statistical significance was determined using two-way ANOVA followed by Tukey’s post-hoc test. *p < 0.05; **p < 0.01; ***p < 0.001; ****p < 0.0001. *AEA* *N*-arachidoyl-ethanolamine, *AG* arachidonoyl-glycerol, *ALA* α-linolenic acid, *DHEA* *N*-docosahexaenoyl-ethanolamine, *DHG* docosahexaenoyl-glycerol, *DPEA* *N*-docosapentaenoyl-ethanolamine, *DPG* docosapentaenoyl-glycerol, *EPEA* *N*-eicosapentaenoyl-ethanolamine, *EPG* eicosapentaenoyl-glycerol, *HS* skin substitute, *LEA* *N*-linoleoyl-ethanolamine, *LG* linoleoyl-glycerol, *ND* not detected, *OEA* *N*-oleoyl-ethanolamine, *OG* oleoyl-glycerol, *PEA* *N*-palmitoyl-ethanolamine, *PG* palmitoyl-glycerol, *PS* psoriatic substitute, *SEA* *N*-stearoyl-ethanolamine, *veh* vehicule.

Total levels of NAEs and MAGs were compared between HS^-^ and PS^-^ epidermis using LC–MS/MS (Supplementary Fig. S1). NAE and MAG total levels were not significantly different between PS^−^ and HS^−^ epidermis (Supplementary Table S1). Additionally, the levels of both n-6 PUFA and n-3 PUFA NAEs and MAGs were not different in PS^-^ compared with HS^-^ (Supplementary Table S2). Levels of SEA were increased in PS^-^ epidermis compared with HS^-^ epidermis (Fig. 2b).

### Long-term ALA administration stimulates the biosynthesis of selected MAGs and NAEs

The treatment of tissue-engineered psoriatic skin substitutes with 10 µM ALA was shown to decrease proliferation and to restore the differentiation of psoriatic keratinocytes to normal^29^. To identify whether ALA treatment modulated the profile of MAGs and NAEs, the effect of long-term treatments mimicking nutritional intervention with 10 µM of ALA on the biosynthesis of NAEs and MAGs was assessed in the psoriatic skin substitute epidermis (Fig. 2a). Treatments with ALA induced an increase in EPEA levels (28.6-fold) and 1/2-DPG (12.3-fold) in PS^ALA+^ (Fig. 2b). The levels of NAEs and MAGs derived from n-6 PUFAs, SFAs and MUFAs were not significantly different in PS^−^ as compared with PS^ALA+^ and therefore treatments with ALA had no effect on epidermal levels of these lipid mediators under our culture conditions (Fig. 2b).

### EPEA decreases the proliferation and increases the differentiation of psoriatic keratinocytes

**Figure 3:**
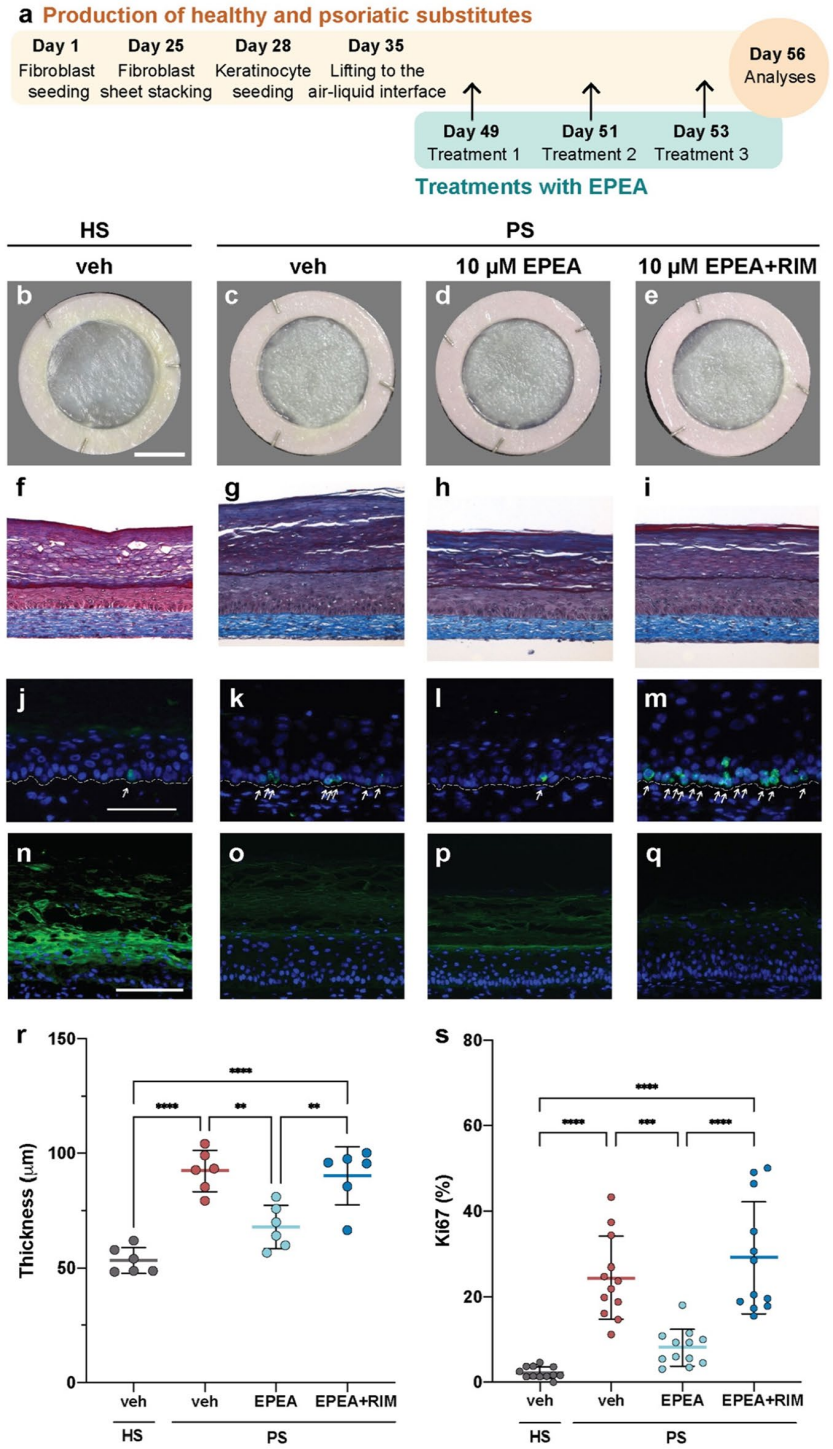
Effect of 10 µM EPEA on psoriatic keratinocyte proliferation and differentiation. (**a**) Schematic overview of skin substitute reconstruction according to the self-assembly method, and treatment with either 10 µM EPEA, 10 µM EPEA + RIM or the vehicle. (**b**–**e**) Macroscopic aspect and (**f**–**i**) histological appearance of cross-sections after Masson’s trichrome staining of the skin substitutes. (**j**–**m**) Detection of Ki67 (green) and (**n**–**q**) filaggrin (green) by immunofluorescence. White arrows indicate Ki67 positive cells. Nuclei were stained with DAPI (blue). The dotted line indicates the dermo-epidermal junction. Scale bars: (**b**–**e**) 1 cm, (**f**–**q**) 100 μm. (**r**) Epidermal thickness quantification from Masson’s trichrome-stained sections. (**s**) Ratio of Ki67 positive cells to the number of total keratinocytes in the basal layer. (N = 3 donors, n = 2 reconstructed substitutes per donor). Statistical significance was determined using one-way ANOVA followed by Tukey’s post-hoc test. *p < 0.05; **p < 0.01; ***p < 0.001; ****p < 0.0001. *EPEA* *N*-eicosapentaenoyl-ethanolamine, *HS* healthy substitute, *PS* psoriatic substitute, *RIM* Rimonabant, *veh* vehicle.

The effect of EPEA on the skin substitutes was further investigated, since it has a higher affinity for the CB_1_ receptor than 1/2-DPG^17^. To assess the biological activity of EPEA, tissue-engineered psoriatic skin substitutes were produced according to the self-assembly method (Fig. 3a). After 49 days of culture, the psoriatic substitutes were treated three times (days 49, 51 and 53) with culture media containing 10 µM EPEA (PS^EPEA+^), 10 µM EPEA and 10 µM rimonabant (RIM) (PS^EPEA+RIM+^) or the corresponding amount of vehicle (PS^−^) (Fig. 3c–e). Healthy substitutes were produced as controls (Fig. 3b). The biological activity of EPEA on psoriatic keratinocyte proliferation and differentiation was identified on day 56 (Fig. 3a). The thickness of the epidermis was significantly decreased in PS^EPEA+^ compared with PS^−^, approximating that of healthy substitutes (Fig. 3f,g,h,r). Moreover, significantly fewer basal keratinocytes were stained with the proliferation marker Ki67 in PS^EPEA+^ compared with PS^−^, closer to the healthy phenotype (Fig. 3j,k,l,s). Taken together, these results show that EPEA treatment decreased psoriatic keratinocyte proliferation. The impact of EPEA in combination with RIM, a CB_1_ receptor antagonist, on psoriatic keratinocyte proliferation was next assessed to determine if the effect of EPEA was mediated through the CB_1_ receptor. Both the epidermal thickness and the number of Ki67 positive cells were not statistically different between PS^EPEA+RIM+^ and PS^-^, showing that in combination with RIM, EPEA did not decrease the proliferation of psoriatic keratinocytes (Fig. 3g,i,k,m,r,s). Indeed, the proliferation of psoriatic keratinocytes even tends to increase in PS^EPEA+RIM+^ as compared with PS^−^, although not significant (Fig. 3k,m,s). Therefore, these results show that EPEA decreased psoriatic keratinocyte proliferation through CB_1_ receptor activation. The impact of EPEA on the expression of a late differentiation marker of the epidermis, namely filaggrin, was then evaluated using immunofluorescence labeling in order to determine whether this lipid mediator also affects epidermal differentiation (Fig. 3n,o,p,q). Interestingly, filaggrin expression of the late differentiation marker was increased in PS^EPEA+^ compared with PS^−^, suggesting increased differentiation of the psoriatic skin substitutes with the treatment (Fig. 3o,p). In contrast, filaggrin levels were not increased in PS^EPEA+RIM+^, thus showing that the effect was CB_1_-dependant (Fig. 3o,q).

### EPEA is incorporated into psoriatic substitutes and reduces psoriatic features and markers of inflammation

**Figure 4:**
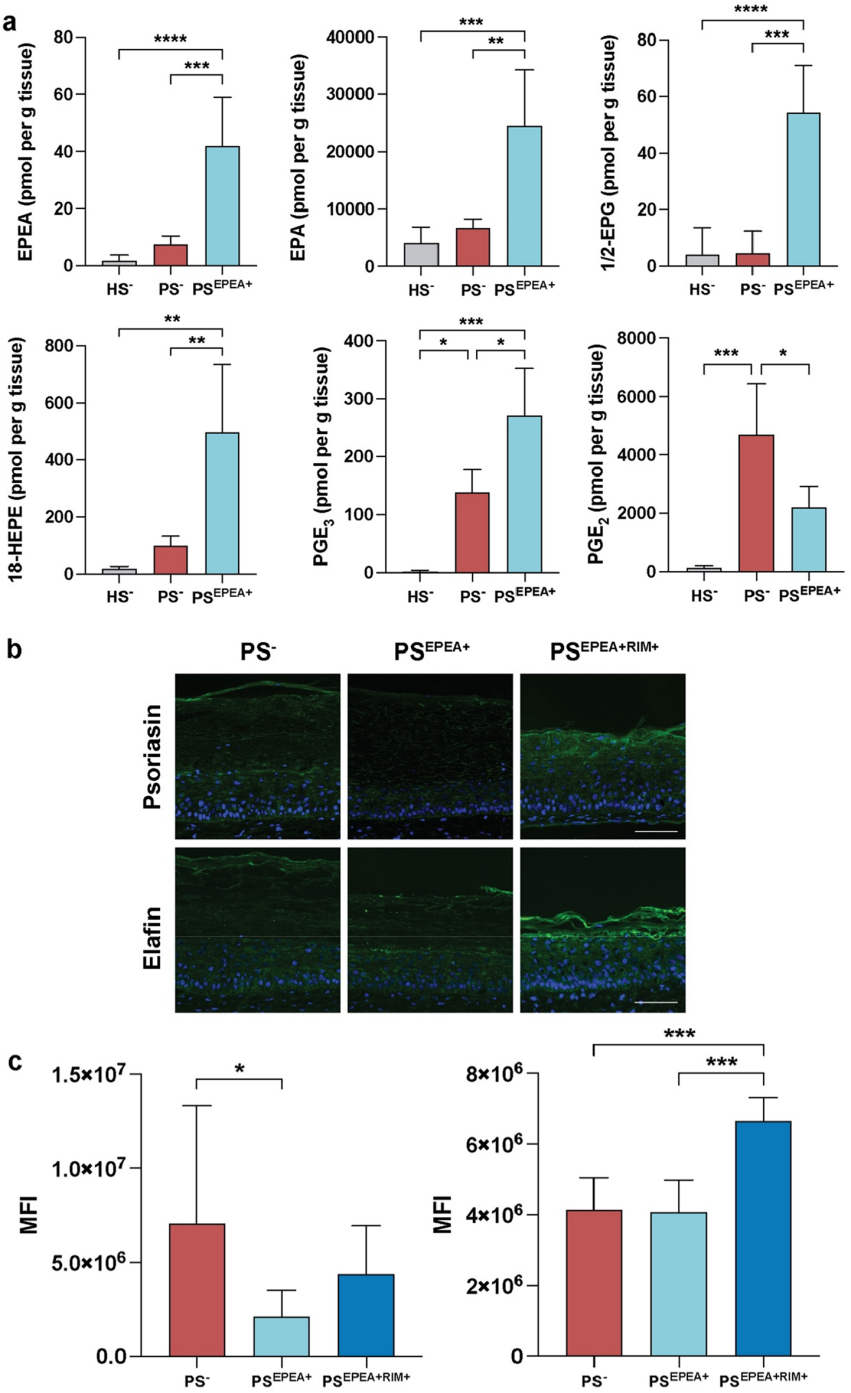
Effect of 10 μM EPEA on inflammation and psoriatic markers. (**a**) Levels of n-3-derived NAEs, various EPA-derived lipid mediators and PGE_2_ in healthy and psoriatic skin substitutes measured using LC–MS/MS (N = 2 donors, n = 2 reconstructed substitutes per donor). (**b**) Expression of psoriasin and elafin (green) in psoriatic substitutes as revealed by indirect immunofluorescence. Nuclei were stained with DAPI (blue). (**c**) Mean fluorescence intensity (MFI) of psoriasin and elafin measured by indirect immunofluorescence. Scale bars: 100 μm. Statistical significance was determined using one-way ANOVA followed by Tukey’s post-hoc test. *p < 0.05; **p < 0.01; ***p < 0.001. *EPEA* *N*-eicosapentaenoyl-ethanolamine, *HS* healthy substitute, *PGE*_*2*_ prostaglandin E_2_, *PS* psoriatic substitutes, *RIM* Rimonabant, *DHEA* N-docosahexaenoyl-ethanolamine, *DPEA* *N*-docosapentaenoyl-ethanolamine, *EPEA* *N*-eicosapentaenoyl-ethanolamine, *HEPE* hydroxy-eicosapentaenoic acid, *HS* healthy substitute, *NAE* *N*-acyl-ethanolamine, *PGE3* prostaglandin E3, *PS* psoriatic substitute, *RIM* Rimonabant.

The ability of EPEA to regulate lipid mediators, inflammation-related proteins and cytokines was next addressed (Fig. 4 and Supplementary Fig. S1). LC–MS/MS analyses were performed to evaluate whether EPEA was efficiently incorporated into PS^EPEA+^ and whether EPEA incorporation further modulated the epidermal lipid profile of PS^EPEA+^. In that regard, treatment with EPEA resulted in increased levels of EPEA in PS^EPEA+^ epidermis compared with PS^-^ epidermis, confirming the efficient uptake of EPEA by psoriatic keratinocytes (Fig. 4a). The levels of various EPA metabolites were also increased in PS^EPEA+^ epidermis, including 15-HEPE (4.5-fold, p-value < 0.05), 18-HEPE (fivefold, p-value < 0.01) and PGE_3_ (twofold, p-value < 0.01) (Fig. 4a). Along these lines, LC–MS/MS analyses revealed lower levels of AA-derived PGE_2_ in PS^EPEA+^ than in PS^−^ (Fig. 4a). Based on indirect immunofluorescence analysis, psoriasin levels were decreased in PS^EPEA+^ compared with PS^−^ (Fig. 4b,c). No significant effects of EPEA on elafin levels were noticed. Finally, the levels of two cytokines secreted by keratinocytes, IL-6 and TNF-α, were assayed in the culture media (supernatant) of the skin substitutes at day 56 by ELISA (Supplementary Fig. S2). The treatments with EPEA had no effect on the secretion of either cytokines as levels of IL-6 and TNF-α were not significantly different between PS^-^ and PS^EPEA+^ (Supplementary Fig. S2).

## Discussion

In the past years, cannabinoid research has been mainly focused on the therapeutic potential of the CB_2_ receptor due to limitations encountered with the targeting of the CB_1_ receptor. Indeed, stimulation of the CB_1_ receptor following systemic administration induced unwanted psychotropic effects. In addition, the antagonism of the CB_1_ receptor with compounds like RIM is efficient as an appetite suppressants for the treatment of obesity, but these compounds were not approved by the Food and Drug Administration in the USA, while being discontinued by the European Medicines Agency (EMEA) after two years, because the potential benefits were not outweighing the risks of severe adverse events linking to these compounds^30^. Noteworthy, the unwanted side effects associated with drugs targeting the CB_1_ receptor inverse agonist or antagonist could be avoided in the treatment of skin diseases using a suitable topical formulation. This prompted us to question whether n-3 PUFA-derived NAEs would be effective for treating psoriasis. In this regard, the present study shows that (1) NAE and CB_1_ receptor signaling could be altered in reconstructed psoriatic skin substitutes, (2) treatments with ALA increased the levels of EPEA and 1/2-DPG in the psoriatic skin substitutes, and (3) EPEA decreased psoriatic keratinocyte proliferation in a CB_1_-dependant manner.

The skin has been reported to contain a fully functional endocannabinoid system^31,32^. However, the endocannabinoidome of psoriatic skin had not been previously investigated. In the present study, psoriatic skin substitutes displayed dysregulated gene expression related with the endocannabinoidome compared with healthy skin substitutes, with decreased mRNA expression in NAE hydrolases (*FAAH2*) and increased mRNA expression in MAG hydrolases (*MAGL*, *ABHD6* and *ABHD12*). The decrease in *FAAH2* expression observed in psoriatic substitutes could be due to reduced epidermal differentiation compared with healthy substitutes, in line with reports showing that *FAAH2* expression increased with differentiation of the keratinocyte cell line HaCaT^31^. Dysregulation of NAE and MAG hydrolases in psoriatic substitutes would have been expected to result in altered NAE and MAG content. This has been documented in other models. For instance, knockdown or inhibition of FAAH in mice led to increased NAE levels^33,34^. Moreover, decreased expression of FAAH1 and FAAH2 was associated with increased levels of NAEs in the skeletal muscle of south Asian as compared with Caucasians^35^. However, the comparison of lipid mediator levels revealed few differences between psoriatic and healthy skin substitutes, with only SEA levels being increased in the psoriatic substitutes. These results therefore suggest high homeostatic stability in the regulation of the endocannabinoid system in the reconstructed skin substitutes. It is also important to mention that only selected NAEs and MAGs were analysed in the present study and that they might not reflect total levels of NAEs and MAGs. Overexpression of the CB_1_ receptor was also reported in psoriatic arthritis and was suggested to contribute to a higher oxidative stress^36^. The CB_2_ receptor is documented as being mainly expressed in immune cells^37^.

Treatments of the psoriatic skin substitutes with ALA induced increased levels of EPEA and 1/2-DPG in the reconstructed skin substitutes, showing an efficient biosynthesis of both NAEs and MAGs. This indicates that the supply of n-3 PUFA does influence the levels of associated NAEs and MAGs. In an animal model, it was reported that a diet rich in AA increased the levels of AEA and 2-AG jejunum, while a diet rich in EPA and DHA decreased the levels of all NAEs in the liver, except EPEA and DHEA^38^. In the brain, a deficiency in n-3 PUFA leads to increased levels of 2-AG, while short-term supplementation with long-chain n-3 PUFAs reduced brain 2-AG levels^39^.

In the present study, EPEA was shown to decrease psoriatic keratinocyte proliferation in a CB_1_-dependent manner. EPEA and DHEA bind to the CB_1_ and CB_2_ receptors although to a lesser extent than AEA^17,40^. In fact, EPEA displayed 4- to 16.5-fold weaker binding to CB_1_ than AEA^41–43^. EPEA and DHEA have however demonstrated anti-proliferative effects on cancer cells via their activation of both CB_1_ and CB_2_ receptors, and the potencies of both could be enhanced by inhibiting the AEA metabolizing enzyme^44^. In addition, it was found that DHEA exerts an anti-seizure action through its activation of the CB_1_ receptor^45^. Finally, DHEA and EPEA were shown to decrease LPS- induced adipocyte IL-6 and MCP-1 levels and that those effects were produced through their liaison to the CB_2_ receptor^46^. Likewise, DHEA was shown to have 8–22.5 times less affinity for CB_1_ receptor than AEA^43,47^. Several studies have shown that the effects of PEA, SEA, OEA, 1/2-OG and 1/2-PG are exerted independently of their binding to the CB_1_ and CB_2_ receptors. Instead, they activate PPARα or other targets such as TRPV1 and the orphan G protein-coupled receptors GPR119 and GPR55^15^. Therefore, the involvement of these other receptors should be further investigated. Although the effect of EPEA has to our knowledge never been studied in healthy and diseased skin, the activation of the CB_1_ receptor with various agonists has been investigated. In the skin, agonists of CB_1_ receptor were reported to induce apoptosis in cancer cell lines by increasing intracellular calcium levels, which induce ER stress and the production of ROS, leading ultimately to mitochondrial dysfunction^48^. In the epidermis, slight to moderate CB_1_ receptor activation may operate as a suppressor of keratinocyte differentiation, whereas its activation through high concentrations of AEA leads rather to anti-proliferative and pro-apoptotic events^49^. Furthermore, the knockout of CB_1_ receptor in a mouse model delayed the recovery of the epidermal impermeable barrier, while the knockout of CB_2_ receptor seemed to accelerate this process^50^. Moreover, the treatment with AEA of normal human epidermal keratinocytes activated with INFγ, and that of immortalized human keratinocytes decreased the production of TNF-α, the IL-12 p40 subunit, the IL-12 p70 subunit and IL-23 by these cells^51^.

## Conclusion

Endocannabinoidome-related mediators, notably EPEA, exhibit anti-inflammatory properties that make them potential treatments for inflammatory diseases. In the present study, tissue-engineered human psoriatic skin displayed altered levels of proteins of the endocannabinoid system. Additionally, the treatment of psoriatic substitutes with ALA was shown to increase the levels of several n-3 PUFA-derived NAEs and MAGs. Along those lines, EPEA was effective in lessening the hyperproliferation of psoriatic keratinocytes, improving epidermal differentiation and reducing levels of inflammation markers in psoriatic skin. Hence, n-3 derived NAEs, and specifically EPEA, represent a promising avenue of treatment for psoriasis.

## Materials and methods

### Production of tissue-engineered human skin substitutes

The institutional review board of the Research Ethics Committee of the CHU de Québec-Université Laval approved the study and informed consent was obtained from all subjects in accordance with the Helsinki declaration. Healthy fibroblasts and keratinocytes were extracted from the skin biopsies (breast reduction) of three Caucasian women aged 18, 46 and 49 years old^52^. Psoriatic fibroblasts and keratinocytes were isolated from 6 mm biopsies taken directly from the psoriatic plaques of three Caucasian men aged 36, 49, and 64 years old^29^. Cells were isolated according to the method based on thermolysin, trypsin and collagenase digestion described elsewhere^53^.

Healthy skin substitutes (HS) and psoriatic skin substitutes (PS) were produced according to the self-assembly method presented elsewhere^29^. Primary human fibroblasts were cultured for 25 days in 6-well plates using Dulbecco’s modified Eagle’s (DME) medium (Gibco, Life Technologies, New York, NY, US) supplemented with 10% Fetal Calf Serum (Wisent Inc., St-Bruno, QC, CAN), 50 mg/ml ascorbic acid (Sigma, Oakville, ON, CAN), 0.06 mg/ml penicillin G (Sigma, Oakville, ON, CAN) and 25 mg/ml gentamicin (Gemini Bio-Products, Sacramento, CA, US). Two of the resulting fibroblast sheets were superimposed and were cultured for 3 days to form a thicker dermal layer. Human primary keratinocytes extracted from the same donor as their matching fibroblasts were then seeded on the surface of the dermal equivalents. The resulting skin substitutes were cultured in submerged conditions for one week in DME mixed with Ham’s F12 medium (3:1) supplemented with 5% FetalClone II serum (Hyclone, Logan, UT, US), 5 mg/ml insulin (Sigma, Oakville, ON, CAN), 0.4 mg/ml hydrocortisone (Galenova, St-Hyacinthe, QC, CAN), 10^–10^ M cholera toxin (Sigma, Oakville, ON, CAN), 10 ng/ml human epidermal growth factor (Ango Inc., San Ramon, CA, US), 60 mg/ml penicillin and 25 mg/ml gentamicin. The HS were then raised to the air–liquid interface and cultured for three additional weeks. Culture media were changed three times a week.

### Treatment of the skin substitutes

For ALA supplementation of the culture media, a stock solution was first produced by dissolving ALA (Sigma, Burlington, ON, CAN) in 99% ethanol (EtOH) (Greenfield Global, Brampton, ON, CAN). The stock solution was then added to the culture media to obtain a final concentration of 10 µM, a concentration selected according to our previous dose–response study^29^. Psoriatic substitutes were produced either with all culture media supplemented with ALA (PS^ALA+^) or with culture media supplemented with the corresponding volume of EtOH (PS^-^).

For endocannabinoid assays, stock solutions were prepared by dissolving eicosapentaenoyl ethanolamide (EPEA) and CB_1_ antagonist Rimonabant (RIM) (Cederlane, Burlington, ON, CAN) in 99% EtOH (Greenfield Global, Brampton, ON, CAN). Psoriatic skin substitutes were produced with regular culture media until they displayed a representative psoriatic phenotype (up to day 49). Psoriatic substitutes were then treated three times (Days 49, 51, and 53) with culture media containing 10 µM EPEA, 10 µM RIM or the vehicle (< 0.01% EtOH).

### Gene expression profiling

Total RNA was isolated from the complete skin substitutes (dermal and epidermal compartments) using the RNeasy Mini Kit (Qiagen, Toronto, CAN), and its quality was determined (2100 Bioanalyzer, Agilent Technologies, Mississauga, CAN) as described^23^. The labeling of Cyanine 3-CTP-labeled targets, their hybridization on a G4851A SurePrint G3 Human GE 8 × 60 K array slide (Agilent Technologies), and data acquisition and analyses were all performed as previously reported^23^ (GSE120464, http://www.ncbi.nlm.nih.gov/geo/query/acc.cgi?acc1⁄4GSE120464). For statistical purposes, microarrays were performed with healthy and psoriatic skin substitutes reconstructed using matching fibroblasts and keratinocytes from additional donors: five healthy donors (aged 18, 22, 23, 38, and 46 years old) and four donors with psoriasis (aged 35, 47, 49, and 64 years old).

### Histologic analysis

Skin substitute biopsies were fixed in HistoChoice solution (AMRESCO, Inc., Solon, OH, US) and embedded in paraffin. Five µm thick sections were cut and were stained with Masson’s trichrome staining. Two substitutes for each of the 3 donors were analyzed (n = 6). Skin sections were observed using a Zeiss microscope equipped with an AxioCam ICc1 camera (Carl Zeiss Meditec, AG, Oberkochen, Germany). The thickness of the dermis and epidermis was measured on the stained sections using ImageJ software (National Institutes of Health, Bethesda, MD, US; http://imagej.nih.gov/ij). A total of 10 measurements on three sections of each biopsy were made.

### Indirect immunofluorescences

Skin substitute biopsies were embedded in Tissue-Tek OCT Compound (Optimum Cutting Temperature, Sakura Finetek, Torrance, CA, US) and stored at − 80 °C until further analysis. Using a Leica cryostat, 5 µm thick sections were sliced and mounted on microscope slides. The skin sections were fixed in cold acetone for 10 min. In a humid chamber protected from light, the skin sections were then incubated with the primary antibodies in PBS with 1% BSA for 1 h, washed, and then incubated with the secondary antibodies in PBS with 1% BSA for 45 min. See Supplementary Table S1 for complete information regarding primary and secondary antibodies. The slides were mounted with mounting media containing DAPI Fluoromount-G (SouthernBiotech, Birmingham, AL, US), which stains the cell nucleus in blue. Skin sections were observed using a Zeiss microscope equipped with an AxioCam HR Rev3 camera (Carl Zeiss Meditec, AG, Oberkochen, Germany).

### LC–MS/MS

The epidermis of the skin substitutes was mechanically separated from the dermis using forceps and scalpels. Skin samples were pulverized into fine powder using a Cryomill MM400 (Retsch^®^, Newtown, PA, US) and suspended in 500 ml of 50 mM Tris–HCl (pH 7) and immediately denatured in one volume of cold (− 20 °C) methanol containing the internal standards. The extraction of epidermal and dermal lipids was performed using the Bligh and Dyers technique, exactly as described in Ref.^54^. Samples were reconstituted in 50 ml of a 50/50 mixture of LC solvent A (H_2_O containing 0.05% acetic acid and 1 mM NH4^+^) and solvent B (acetonitrile/H_2_0, 95/5, v/v, with 0.05% acetic acid and 1 mM NH4^+^). 40 ml of the latter was injected onto a RP/HPLC column (Kinetex C8, 150 × 2.1 mm, 2.6 mm, Phenomenex) and lipids were separated using the same LC program as described previously^55^. Quantification was done by generating calibration curves and analyzed using pure standards on the LC–MS/MS system three times. The slope was then calculated using the ratio between the peak areas of the compound and its standard (Supplementary Table S1). Because of the acyl migration from *sn*-2- to *sn*-1(3) naturally occurring in MAGs, we present the data as the combination of MAG isomers (1/2-MAG).

### ELISA assays

Culture supernatants were collected on day 56 and stored at −80 °C until use. The secreted levels of IL-6 and TNF-α were assayed using an IL-6 Human ELISA Kit (KHC0061, ThermoFisher Scientific, Waltham, MA, USA) and a TNF-α Human ELISA Kit (KHC3011, ThermoFisher Scientific, Waltham, MA, USA) respectively. All procedures were performed following the manufacturer’s instructions. The absorbance of IL-6 and TNF-α were measured at 450 nm using a SpectraMax Plus 384 microplate spectrophotometer (Molecular Devices, San Jose, CA, USA).

### Statistical analysis

Data were analyzed using Two-way ANOVA followed by Tukey’s post-hoc test and expressed as means ± standard deviation. A p-value < 0.05 conferred statistical significance. All calculations were performed with Prism version 7 software (Graphpad Software, La Jolla, CA).

## Supplementary Information

Supplementary Information.

## Data Availability

All microarray data presented in this study comply with the Minimum Information About a Microarray Experiment requirements. The gene expression data have been deposited in the National Center for Biotechnology and Information’s Gene Expression Omnibus (http://www.ncbi.nlm.nih.gov/geo/) and are accessible through Gene Expression Omnibus Series accession number (GSE120464, http://www.ncbi.nlm.nih.gov/geo/query/acc.cgi?acc1⁄4GSE120464).
