# Supplementary material for: N-eicosapentaenoyl-ethanolamine decreases the proliferation of psoriatic keratinocytes in a reconstructed psoriatic skin model
Source: Sci Rep. 2023 Jul 26;13:12113. doi: 10.1038/s41598-023-39185-4 (PMC10371979; doi:10.1038/s41598-023-39185-4)
Supplement: Supplementary file 1 — Supplementary Information. [file 41598_2023_39185_MOESM1_ESM.docx]

**Supplementary Material**

**Table S1: Antibodies used for immunofluorescence analyses**

| **Name of Antibody** | **Source** | **Dilution** | **Catalog No.** | **Supplier** | **Supplier Location** |
| --- | --- | --- | --- | --- | --- |
| Anti-mouse Alexa 488 | Goat | 1:1400 | A11001 | Life | Eugene, OR |
| Anti-rabbit  Alexa 488 | Goat | 1:1200 | A1103 | Life | Eugene, OR |
| Elafin | Rabbit | 1:100 | Ab4774 | Abcam | Cambridge, MA |
| Filaggrin | Mouse | 1:500 | SC-66192 | Santa Cruz Biotechnology | Heidelberg, Germany |
| Ki67 | Mouse | 1:300 | 556003 | BD Biosciences | San Diego, CA |
| Psoriasin | Rabbit | 1:300 | Ab83534 | Abcam | Cambridge, MA |
| Transglutaminase 1 | Rabbit | 1:300 | 12912-3-AP | Cedarlane | Burlington, Canada |

**Table S2. Total Levels of NAEs and MAGs in healthy and psoriatic skin substitutes after ALA treatments.**

|  | | **Mean ±SD (pmol/g of tissue)** | | |  | **P-value** | |
| --- | --- | --- | --- | --- | --- | --- | --- |
| **Bioactive lipids** | | **HS^-^** | **PS^-^** | **PS^ALA+^** |  | **PS^-^ vs PS^ALA+^** | **PS^-^ vs HS^-^** |
|  | Total NAEs | 18059 ± 8714 | 23434 ± 10425 | 18414 ± 7792 |  | NS | NS |
|  | Total n-3 NAEs | 7.442 ± 2.906 | 4.292 ± 3.521 | 47.67 ± 35.09 |  | 0.0360 | NS |
|  | Total n-6 NAEs | 433.1 ± 139.9 | 337.6 ± 51.62 | 435.6 ± 130.6 |  | NS | NS |
|  | Total SFA and MUFA NAEs | 17618 ± 8610 | 23092 ± 10398 | 17931 ± 7651 |  | NS | NS |
|  | Total MAGs | 85522 ± 21270 | 52214 ± 18798 | 40127 ± 21740 |  | 0.0445 | NS |
|  | Total n-3 MAGs | 210.3 ± 155.0 | 399.5 ± 116.8 | 1419 ± 766.3 |  | NS | 0.0282 |
|  | Total n-6 MAGs | 3479 ± 2574 | 3488 ± 1250 | 3328 ± 1579 |  | NS | NS |
|  | Total SFA and MUFA MAGs | 81832 ± 20075 | 48327 ± 17474 | 37231 ± 19639 |  | NS | NS |
|  | EPEA | 2.495 ± 2.087 | 1.231 ± 0.318 | 35.231 ± 33.795 |  | NS | 0.0092 |
|  | DHEA | 4.950 ± 1.212 | 3.060 ± 3.273 | 12.438 ± 8.226 |  | NS | NS |
|  | LEA | 405.863 ± 128.938 | 301.914 ± 53.279 | 322.531 ± 173.104 |  | NS | NS |
|  | AEA | 27.230 ± 14.497 | 35.645 ± 10.693 | 68.690 ± 74.762 |  | NS | NS |
|  | PEA | 3239.398 ± 1590.636 | 1570.453 ± 673.182 | 884.676 ± 527.979 |  | NS | NS |
|  | OEA | 9357.315 ± 4058.082 | 4841.281 ± 1448.029 | 6708.243 ± 4529.641 |  | NS | NS |
|  | SEA | 5021.438 ± 3075.666 | 16680.360 ± 8507.989 | 10338.169 ± 4098.643 |  | 0.0008 | NS |
|  | 1/2-EPG | 5.967 ± 11.935 | 33.906 ± 9.619 | 159.945 ± 65.101 |  | NS | NS |
|  | 1/2-DPG | 139.172 ± 87.616 | 80.905 ± 34.809 | 992.747 ± 783.973 |  | NS | 0.0001 |
|  | 1/2-DHG | 65.196 ± 56.074 | 284.642 ± 86.580 | 266.657 ± 85.587 |  | NS | NS |
|  | 1/2-LG | 2929.705 ± 2262.649 | 2155.287 ± 835.924 | 2711.371 ± 1534.934 |  | NS | NS |
|  | 1/2-AG | 549.474 ± 319.569 | 1333.079 ± 539.524 | 616.559 ± 598.092 |  | NS | NS |
|  | 1/2-PG | 16387.083 ± 3289.947 | 9903.221 ± 3417.547 | 14656.913 ± 7275.696 |  | NS | NS |
|  | 1/2-OG | 20079.818 ± 12122.743 | 38423.299 ± 14465.388 | 22573.843 ± 14773.886 |  | NS | NS |

Abbreviations: MAG, monoacylglycerol; MUFA, monounsaturated fatty acid; NAE, *N*-acylethanolamine; NS, not significant; SD, standard deviation; SFA, saturated fatty acid.

**
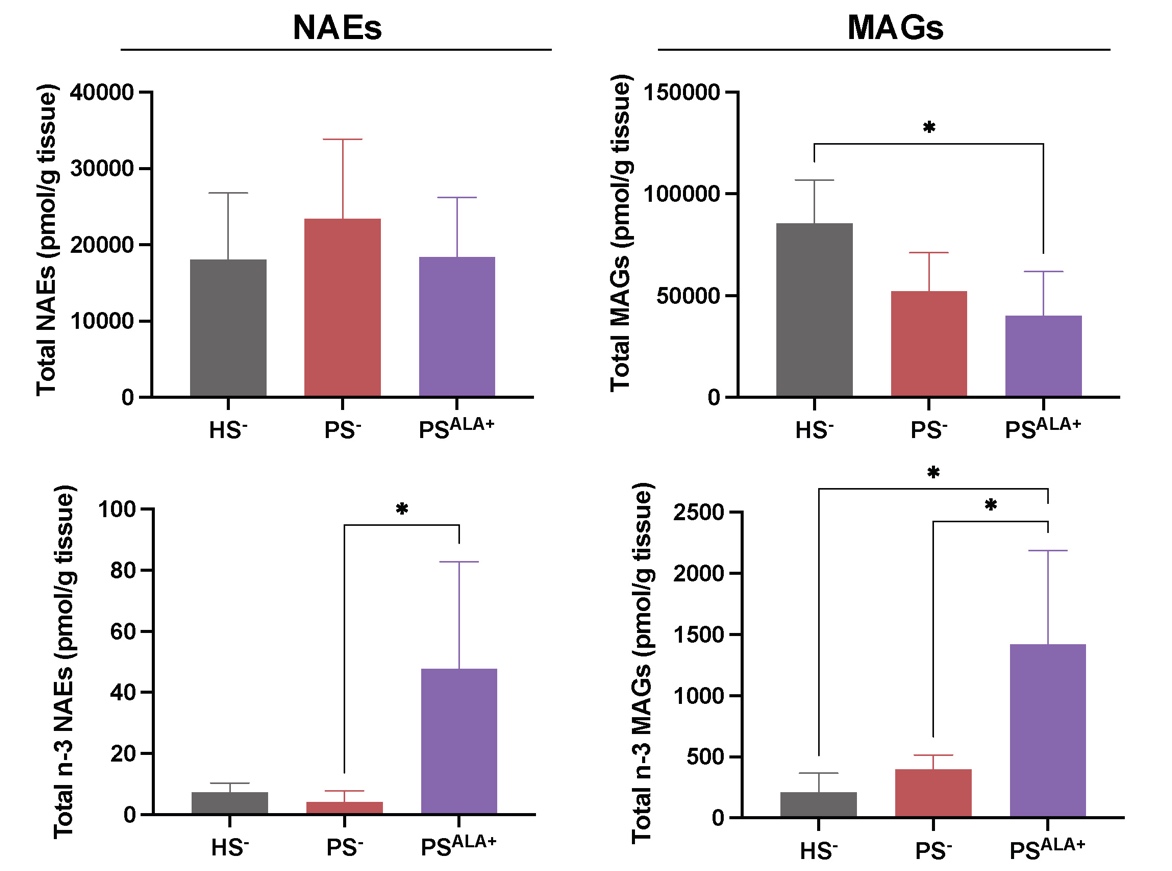
**

**Figure S1. Levels of total NAEs and MAGs in healthy and psoriatic skin substitutes treated with either 10 μM ALA or vehicle.** Levels of NAEs and MAGs were determined by LC-MS/MS. Statistical significance was determined using one-way ANOVA followed by Tukey’s post-hoc test. *p<0.05.


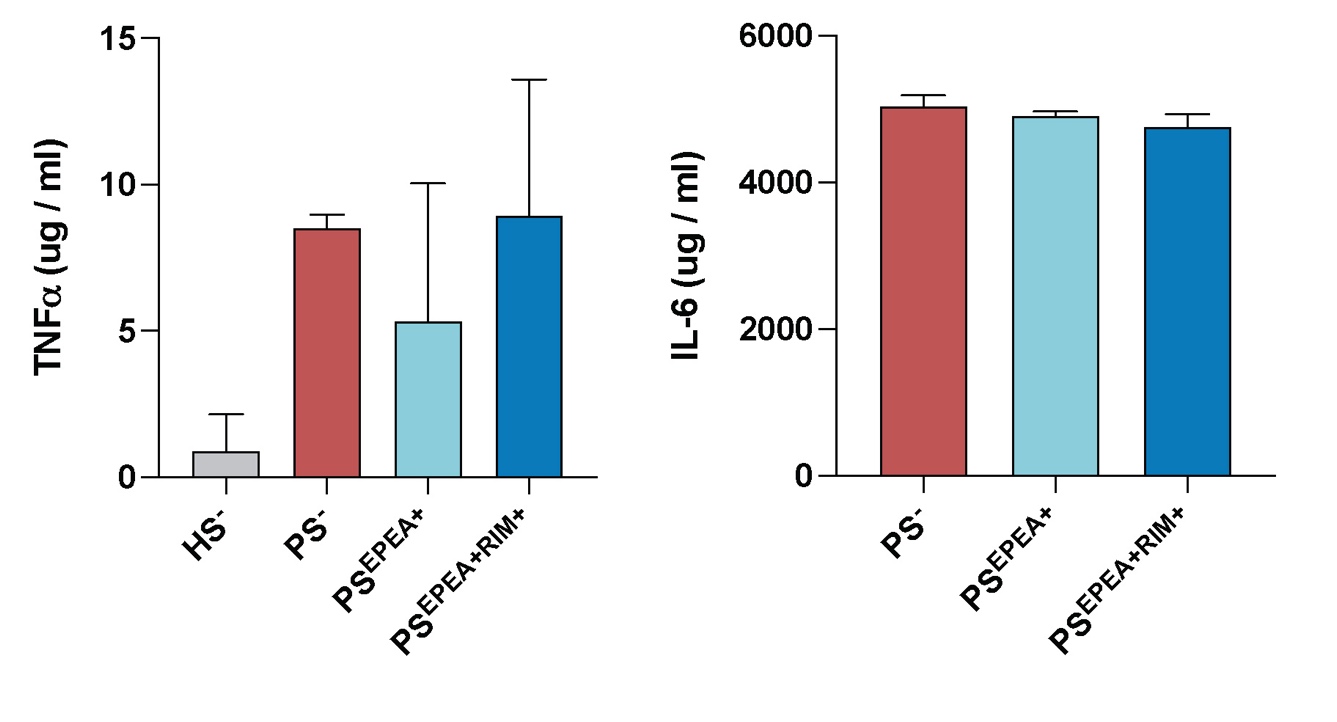


**Figure S2. Levels of TNFα and IL-6 released in the culture media of psoriatic skin substitutes treated with either 10 μM EPEA, or vehicle.** Cytokine were quantified by ELISA. Statistical significance was determined using one-way ANOVA followed by Tukey’s post-hoc test.
